# Pre-cultivation with Selected Prebiotics Enhances the Survival and the Stress Response of *Lactobacillus rhamnosus* Strains in Simulated Gastrointestinal Transit

**DOI:** 10.3389/fmicb.2017.01067

**Published:** 2017-06-14

**Authors:** Mariantonietta Succi, Patrizio Tremonte, Gianfranco Pannella, Luca Tipaldi, Autilia Cozzolino, Rossana Romaniello, Elena Sorrentino, Raffaele Coppola

**Affiliations:** ^1^Dipartimento Agricoltura, Ambiente e Alimenti, Università degli Studi del MoliseCampobasso, Italy; ^2^Scuola di Scienze Agrarie, Forestali, Alimentari ed Ambientali, Università degli Studi della Basilicata, viale dell'Ateneo LucanoPotenza, Italy

**Keywords:** probiotics, prebiotics, stress proteins, *Lactobacillus rhamnosus*, survival, starvation

## Abstract

In our study, we dwelled upon combinations of lactobacilli/prebiotics, considering four different strains belonging to the *Lactobacillus rhamnosus* species, including *Lactobacillus rhamnosus* GG (LGG), and different prebiotics often found in commercial synbiotic products, such as inulin, lactulose and polyols mannitol and sorbitol. In the first step of the research, the survival, the growth kinetic parameters and the protein expression of *Lb. rhamnosus* strains cultivated in presence of the different prebiotics as a unique carbon source were evaluated. In the second step, the influence of pre-cultivation in medium added of metabolizable prebiotics on the strains survival to simulated gastrointestinal (GI) transit, assayed without prebiotics addition, was estimated. Our results showed that the presence in the medium of certain low fermented prebiotics, specific for each strain, represents a stress factor that significantly affects the growth of *Lb. rhamnosus* strains, inducing the up-regulation of several proteins. In detail, all added prebiotics used as unique carbon source caused a growth retard compared with glucose, as testified by increased values of the lag phase and decreased values of the μmax. Mannitol evidenced intermediate μmax values between those registered with glucose and those detected with the other assayed prebiotics. Moreover, the cultivation with prebiotics induced the over expression of 7 protein bands. Interestingly, we found a correlation between the up-regulation of two specific stress proteins, called P4 (ATP-binding subunit Clpx) and P7 (GrpE), and the death kinetic parameters (resistance and cells viability) registered during the simulated GI transit of strains pre-cultivated with specific, low fermented prebiotics. Specifically, the highest resistance and gastric-vitality scores were highlighted for the strain AT195 when pre-cultivated in presence of sorbitol. Conversely, the lowest values were found in the case of DSM20021 pre-cultivated with mannitol. Among the up-regulated stress proteins, P7 resulted involved in the response to the starvation. Finally, it is possible to conclude that the pre-cultivation with certain prebiotics as a unique carbon source represents a strain-specific, sub-lethal stress able to enhance the resistance of *Lb. rhamnosus* strains and consequently their viability under simulated GI transit.

## Introduction

The group of lactic acid bacteria includes several facultatively hetero-fermentative species able to colonize different environmental habitats, including the gastrointestinal tract (GIT), plant materials and foods, such as cheese and dairy products (Succi et al., 2005; Ceapa et al., 2015). Among them, different lactobacilli have a reputed Generally Recognized as Safe (GRAS) status (Kleerebezem and Hugenholtz, 2003). The health-promoting properties of specific strains belonging to *Lactobacillus* spp. have led to their application in products that are marketed as probiotic foods or probiotic pharmaceutical preparations (Succi et al., 2014). It is generally acknowledged that the beneficial effects of probiotics are linked to their ability to survive typical stresses encountered during the storage and the passage through the GIT (Burgain et al., 2011). However, a considerable loss of probiotic vitality is expected. Hence, various approaches were used to improve their resistance to processing and oral administration, and a great attention was paid to protective methods. In the last years, the possible protective role of some prebiotics was also verified. This approach is of great interest, since in this way prebiotics could be used for combined purposes: (i) for their historical role as non-digestible food ingredients that beneficially affect the host by selectively stimulating the growth or/and activity of one/or a limited number of bacteria in the colon (Gibson and Roberfroid, 1995); and (ii) as protective agents against various environmental stresses, comprising those encountered during the GIT. In this field, different types of starch, including modified starches, were used to protect probiotics (Avila-Reyes et al., 2014; Cheow et al., 2016). Moreover, the possible role of prebiotic polyols as delivery vehicles for probiotic bacteria was assessed (Anal and Singh, 2007; Harel and Kohavi-Beck, 2015). Several Authors (Avila-Reyes et al., 2014; Soukoulis et al., 2014) have shown that the use of inulin improves the viability and the stability of *Lactobacillus rhamnosus* GG (LGG), one of the most extensively studied probiotic strain (Saxelin, 1997; Andersson et al., 2001) with thorough clinical documentation (Szajewska and Mrukowicz, 2001; Caramia, 2003; Doron et al., 2005; Viljanen et al., 2005; Kankainen et al., 2009; Aureli et al., 2011; Koskenniemi et al., 2011). These last evidences led the pharmaceutical and the food industry to the development of commercial products with the combined presence of inulin and *Lb. rhamnosus* strains.

To date, if on the one hand the effect of prebiotics on the implementation of probiotic viability in the colon is widely shared, on the other hand their protective role against stresses that affect probiotics during the GI transit is poorly investigated. The mechanisms about how certain non-digestible substances are metabolized by beneficial microbes remain largely unknown (Goh and Klaenhammer, 2015). Genome sequencing of probiotic lactobacilli revealed a versatile carbohydrate metabolic gene repertoires dedicated to the catabolism of various oligosaccharides. Several Authors highlighted a significant change in the gene-products and in the enzymatic patterns when prebiotics are assimilated by *Lactobacillus* strains (Saulnier et al., 2007; Hussain et al., 2009). Noteworthy, it is known that *Lb. rhamnosus* genome is predicted to bring a large number of carbohydrate transport and utilization genes that display substantial variations among strains (Douillard et al., 2013b). The genetic architecture of *Lb. rhamnosus* supports its high versatility and adaptability to multiple niches. In fact, strains belonging to this species are able to grow in environments rich in lactose, such as dairy products, or in plant-associated environments rich in sucrose, trehalose, maltose, cellobiose, raffinose, starch, inulin, and fructosans, as well as in the intestinal tract, containing diet-derived and host-derived carbohydrates, such as fucose, hexosamines, mannose, and galactose (Flint et al., 2007; Zalán et al., 2010).

The study reported herein focused the attention on the ability of four *Lb. rhamnosus* strains to grow with different prebiotics represented by polyols, lactulose, and inulin. Moreover, the effect of pre-cultivation in presence of prebiotics on the resistance to GI stress conditions was evaluated in simulated GI solutions not added of prebiotics.

## Materials and methods

### Bacterial strains and prebiotics

In this study, four *Lb. rhamnosus* strains were used. Two strains (*Lb. rhamnosus* AT194 and *Lb. rhamnosus* AT195), previously isolated from Parmigiano Reggiano cheese (Coppola et al., 2005), were from the Food Microbiology Culture Collection of the DIAAA (Dept. of Agricultural, Environmental and Food Science, University of Molise). The type strain DSM20021 was provided by the Leibniz Institute DSMZ-German Collection of Microorganisms and Cell Cultures (Braunschweig, Germany), and the commercial strain *Lactobacillus* GG was previously isolated from a pharmaceutical preparation (Valio LTD, Helsinki, Finland), as described by Succi et al. (2014). Strains were maintained at −80°C in skim milk (Succi et al., 2007) and propagated twice in MRS broth (Oxoid, Milan, Italy) at 37°C prior their use.

D-sorbitol (extra pure for microbiology), D-mannitol (≥ 99.0% purity) and lactulose (≥ 98.0% purity) were acquired from Sigma-Aldrich (Italy); inulin with a purity of 92% and a DP between 3 and 60 (Fibruline instant) was acquired from Cosucra (Warcoing, Belgium). Each prebiotic was dissolved in sterile distilled water in order to obtain a 25% (w/v) stock solution.

### Growth of *Lb. rhamnosus* in presence of prebiotic

*Lactobacillus rhamnosus* strains grown in MRS broth (Oxoid, Milan, Italy) at 37°C were taken in the mid-exponential phase and centrifuged at 7,500 rcf for 15 min at 4°C (Centrifuge Eppendorf, 5804R). The pellet was washed 2 times with 1X phosphate buffer (1X PBS) and 1% resuspended in Erlenmeyer flasks containing 500 mL of sterilized modified MRS (final pH 6.2) prepared following the standard formula (MRS broth, Oxoid, Milan, Italy) with glucose and citrate omitted, and added of 10 g/L (final concentration) of each filter-sterilized prebiotic (Filter Unit Red 0.22-μm pore size; Schleider&Schuell, Dassel, Germany). Filter-sterilized glucose (Sigma-Aldrich, Italy) at the same concentration was used for comparative purposes. The growth was assessed by plate counts on MRS agar (Oxoid) at regular time intervals. Two replicates were made for each experiment. The growth kinetic parameters were estimated with the D-model of (Baranyi and Roberts, 1994) using the excel add-in DMFit v.3 (Baranyi and Le Marc, 1996). In detail, maximum specific grow rate (μ*max*), lag phase, initial load values (*y_0*) and final load values (*y_end*) were evaluated. Moreover, the load increase was evaluated as reported by the Equation 1.

(1) Δ_Y=y_end−y_0

Three independent experiments were performed and the results were reported as average.

### Cell protein extraction and characterization

Cell proteins were extracted by *Lb. rhamnosus* strains when growth was detected, that is, in modified MRS broth with glucose or with specific prebiotics. For this purpose, a slightly modified version of the method described by Tremonte et al. (2016) was used. Briefly, cells were collected by centrifugation as described above in the middle of the exponential phase. Cells were washed three times with Tris-HCl (50 mM, pH 7.5), standardized at an OD_600_ value of 1.0 and re-suspended in a lysis buffer (Tris-HCl 50 mM, lysozyme 2 mg/mL, mutanolysin 50 U, protease inhibitor cocktail 1X, pH 7.5). Eight glass beads (Ø 0.4 mm) were added to each cellular suspension (140 μL), then suspensions were vortexed (3 min), incubated for 2 h at 37°C, and sonicated for 5 min with an ultrasonic homogenizer (100 W power, 100% amplitude, 0.8 cycle; Labsonic M, Sartorius, Italy) using a probe of 0.5 mm diameter. After centrifugation at 17,500 rcf for 30 min at 4°C, the pellet was discarded and the supernatant (lysis buffer), containing the protein extract, was subjected to the Bradford-based protein assay kit (Bradford protein assay, Bio-Rad, Italy) to determine the protein concentration. Bovine serum albumin was used as a standard. Supernatants were stored at −20°C and then were analyzed by SDS-PAGE using an electrophoretic system as described by Tremonte et al. (2007, 2010). Bands were visualized using a G250 staining kit (Biorad, Italia). The mobility of individual proteins was calculated and the protein profile of the strains was compared. A protein standard with molecular weight ranging from 74.6 to 14.4 Kda (Amersham biosciences) was used. The reproducibility of the SDS-PAGE was estimated by loading cell protein extracts from each strain in two independent trials performed on two gels in triplicate. The relative error (*E*) for each band in each gel was calculated as reported in the Equation 2.

(2) $$E=\left(\frac{R\text{f}-R\text{fm}}{R\text{fm}}\right)100$$

where *R*f is the distance of a protein band from the top of the separating gel and *R*fm is the mean *R*f for the band obtained in different gels (Di Luccia et al., 2016).

SDS profiles were analyzed by QuantityOne image software (Biorad, Italy). The protein bands were enumerated and their volume was calculated. The SDS profiles of proteins from strains cultivated in presence of prebiotics were compared with those from the same strains cultivated in presence of glucose. An over expression of protein bands at a threshold ≥ 2 was considered significant (*P* < 0.05). A new index (HPS_score) representing the over-expression of two specific protein bands in presence of prebiotics was estimated and calculated as described in the Equation 3.

(3) $$\text{HSP}\_\text{score}=\frac{(VP4+VP7)prebiotic}{(VP4+VP7)glucose}$$

where *VP4* indicates the volume of the protein arbitrarily labeled as P4, having a molecular weight of about 45.7 kDa, and *VP7* indicates the volume of the protein arbitrarily labeled as P7, with a molecular weight of about 21.8 kDa.

### Effect of pre-cultivation in prebiotics on *Lb. rhamnosus* survival in simulated gastro-intestinal transit

Strains of *Lb. rhamnosus* were pre-cultivated as reported above in presence of glucose or in presence of the prebiotics which allowed their growth. Cells were collected by centrifugation (7,500 rcf at 4°C for 15 min) in the middle of the exponential phase and washed twice with sterile physiologic solution. Cells were aseptically transferred into sterile physiologic solution at a concentration of about 10 Log CFU/mL (cells stock solution, CSS). The simulated GI transit was carried out according to Succi et al. (2014) with some modifications. Specifically, to simulate the gastric transit, 10 mL of each CSS were added to 100 mL of a sterile electrolyte solution (6.2 g/L NaCl, 2.2 g/L KCl, 0.22 g/L CaCl_2_, 1.2 g/L NaHCO_3_) plus lysozyme (Sigma-Aldrich L6876) 0.1 g/L, and pepsin from porcine gastric mucosa (Sigma-Aldrich P7012) 3.0 g/L, with final pH adjusted to 2.0 (adding 1.0 Mol/L HCl). The suspension was incubated at 37°C for 2 h. To simulate the intestinal transit, the pH of the suspension used for the gastric transit was subsequently adjusted to 7.5 with a sodium bicarbonate saturated solution and 30 mL of a sterile electrolyte solution containing bile salts (bovine bile, Sigma-Aldrich B3883) at a final concentration of 4.5 g/L and pancreatin from porcine pancreas (Sigma-Aldrich P7545) at final concentration of 1 g/L were added. The suspension was incubated in anaerobic conditions at 37°C for 5 h. For each CSS two independent experiments were carried out. At regular time intervals (15 min during the simulated gastric transit and 30 min during the simulated intestinal transit), an aliquot of cultures was recovered from each batch and enumerated by plate counts on MRS agar (Oxoid) after incubation at 37°C for 72 h under anaerobic conditions (AnaeroGen, Oxoid). Two replicates were made for each experiment. The experimental data were used to estimate the death kinetic parameters through the D-model using the excel add-in DMFit v.3 (Baranyi and Roberts, 1994). On the basis of the death kinetic parameters, three indices were calculated as seen in the Equations 4–6.

(4) $$\text{Resistance}\_\text{score}=\frac{Shoulder\_prebiotic\;}{Shoulder\_glucose}$$

where *Shoulder_prebiotic* and *Shoulder_glucose* represent the Shoulder length (h) detected in presence of a specific prebiotic or in presence of glucose, respectively.

(5) $$\text{Vitality}\_\text{score}\_\text{GT}=\frac{yend\_GT\_prebiotic}{yend\_GT\_glucose}$$

where *yend_GT* indicates the microbial values (Log CFU/mL) of strains pre-cultivated in presence of prebiotics or in presence of glucose at the end of the simulated gastric transit (GT)

(6) $$\text{Vitality}\_\text{score}\_\text{GIT}=\frac{yend\_GIT\_prebiotic}{yend\_GIT\_glucose}$$

where *yend_GIT* indicates the microbial values (Log CFU/mL) of strains pre-cultivated in presence of prebiotics or in presence of glucose at the end of the entire simulated gastrointestinal transit (GIT).

### Statistical analyses

Statistical analyses were performed following the approach used by Tremonte et al. (2017). In detail, kinetic parameters, microbial count levels and volume of protein bands were analyzed by a General Linear Model based on ANOVA (IBM SPSS Statistics 21). The *post-hoc* Bonferroni test was used for pairwise comparison. Statistical significance was attributed to *P*-values of <0.05. Statistical data were expressed as mean ± standard deviation.

## Results

### Growth of *Lb. rhamnosus* in presence of different prebiotics

**Figure 1:**
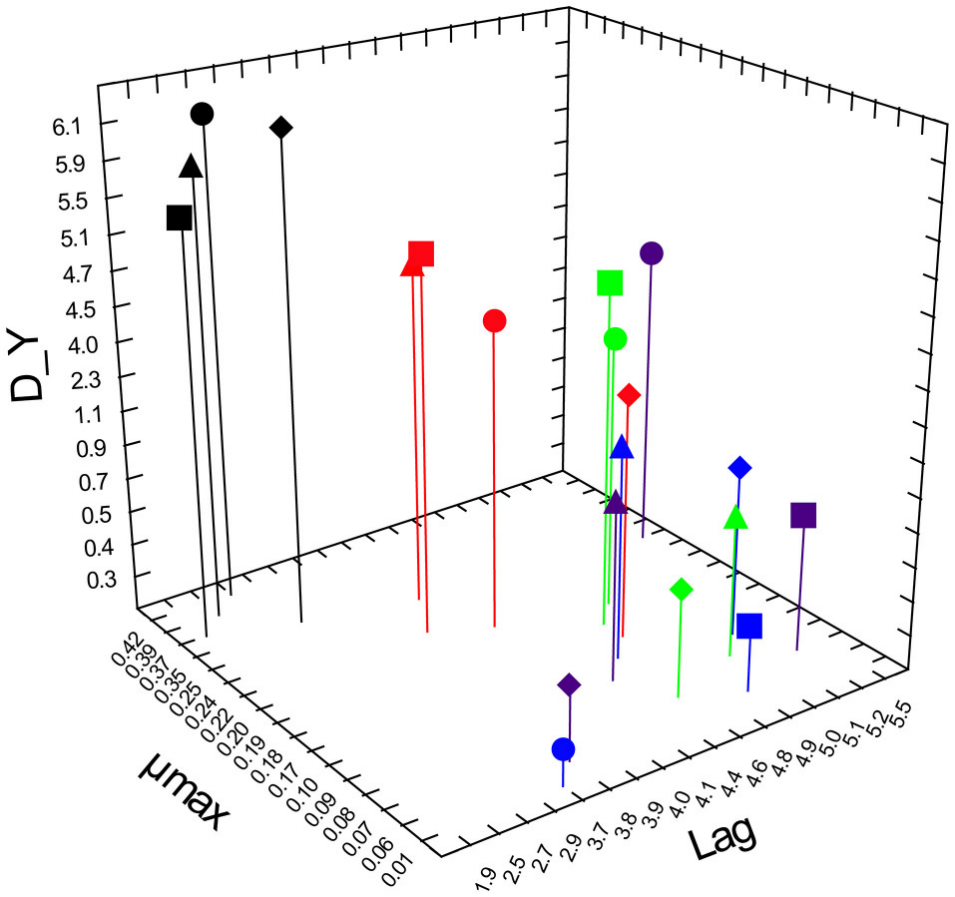
3D Scatterplot representing growth kinetic parameters (Lag phase, Lag; maximum specific growth rate, μmax; increase in microbial load, D_Y) of four *Lb. rhamnosus* strains. AT194 (triangle), AT195 (circle), LGG (square) and DSM20021 (diamond) were cultivated in presence of glucose (black), inulin (blue), lactulose (purple), mannitol (red), or sorbitol (green).

Growth kinetics of *Lb. rhamnosus* in presence of glucose or prebiotics are illustrated in Figure 1. The four assayed strains showed similar parameters when cultivated in presence of glucose (control condition, CC), with no significant differences (*P* > 0.05) in the maximum specific growth rate values (μmax). In CC, the lowest lag phase (1.9 h) was appreciated for *Lb. rhamnosus* GG. Conversely, the statistical analysis highlighted that the different prebiotics affected the growth parameters of tested strains. In detail, all prebiotics caused an increase in the lag phase and a decrease in the μmax, with mannitol showing intermediate μmax values between those registered in CC and those detected with the other assayed prebiotics. Moreover, strains showed different growth capabilities when cultivated with mannitol. In fact, AT195, AT194, and GG had Δ_y values comprised in the range 4.5–5.1, that is, about 2-fold higher than that exhibited by the type strain DSM20021 (about 2.3).

As far as the other prebiotics are concerned, sorbitol allowed the growth of only the strains GG and AT195, while lactulose supported only the growth of AT195. A moderate growth in presence of inulin was detected only for the strain AT194.

### Cell proteins in presence of prebiotics

On the basis of previous results, the cell proteins of *Lb. rhamnosus* strains cultivated in presence of glucose as carbon source or in presence of prebiotics allowing their growth were quantitatively analyzed by the SDS-PAGE (data not shown). The image analysis of gels revealed that the relative error for each band in each gel was <1%. The SDS-profiles of strains cultivated in CC were characterized by 36 main bands with molecular weights (MW) ranging from 10 to 100 kDa. By analyzing the volume values, no significant differences (*P* > 0.05) were detected among the protein bands of all the strains cultivated in CC. Contrarily, an over expression of several protein bands at a threshold ≥ 2 was observed when the strains were cultivated in presence of prebiotics usable as carbon source (Table 1). In that case, significant differences were detected depending on the different strains and on the diverse assayed prebiotics. Specifically, respect to the CC all the strains cultivated with metabolized prebiotics always evidenced the over-expression of 3 protein bands, arbitrarily labeled as P1, P2, and P3. Except for P2 (experimental MW of 91.5 kDa), which resulted unidentified, the other two protein bands had a MW matching with proteins involved in the metabolism of *Lb. rhamnosus* GG (Koponen et al., 2012) or deposited in the *Lb. rhamnosus* GG genomic database (GenBank accession no. FM179322). In particular, the molecular weight of bands P1 and P3 matched with Alanyl-tRNA syntetase (experimental MW of 97.4 kDa; theoretical MW 97.4 kDa) and with Protein traslocase subunit secA (experimental MW of 89.5 kDa; theoretical MW 89.5 kDa), respectively. As stated above, the over-expression degree of P1, P2 and P3 was always detected at a threshold ≥ 2 respect to CC, but it was more or less pronounced as affected by the prebiotic tested and by the strain used. For instance, mannitol always led to the highest over-expression of P1, and, only in GG, also of P2. Sorbitol caused the highest over-expression of P3 in GG and in AT195, whilst lactulose induced the uppermost expression of P2 in AT195.

**Table 1:** Volume, estimated by QuantityOne image software (Biorad), of 7 protein bands recorded in the electrophoretic profile of *Lb. rhamnosus* strains cultivated in MRS broth containing different prebiotics as carbon source.

**Strain**	**Carbon source**	P1 97.4 kDa Alanyl-tRNA synthetase^*^	P2 91.5 kDa Undefined^*^	P3 89.5 kDa Protein translocase subunit secA^*^	P4 45.7 kDa ATP-binding subunit Clpx^*^	P5 29.6 kDa Dihydroorotate dehydrogenase^*^	P6 26.9 kDa Triosephosphate isomerase^*^	P7 21.8 kDa GrpE protein^*^
DSM20021	Glucose	140.1 ± 10.3^a^^**^	100.3 ± 11.1^a^	19.2 ± 2.4^a^	141.4 ± 10.8^a^	32.8 ± 4.5^a^	142.4 ± 16.3^a^	41.1 ± 3.2^a^
	Mannitol	560.4 ± 22.7^b^	210.6 ± 18.2^b^	67.2 ± 5.2^b^	127.2 ± 10.2^a^	36.1 ± 3.9^a^	199.3 ± 15.5^a^	63.9 ± 4.1^a^
GG	Glucose	137.1 ± 12.1^a^	93.5 ± 10.3^a^	18.8 ± 2.3^a^	143.2 ± 13.3^a^	31.4 ± 3.4^a^	153.1 ± 14.9^a^	42.6 ± 3.9^a^
	Mannitol	1039.7 ± 23.4^c^	375.9 ± 17.8^c^	216.6 ± 16.4^c^	325.8 ± 17.2^b^	42.3 ± 2.1^a^	159.8 ± 19.2^a^	119.9 ± 8.6^b^
	Sorbitol	978.5 ± 35.7^c^	256.4 ± 20.3^b^	550.3 ± 21.1^d^	132.4 ± 10.1^a^	178.9 ± 7.2^b^	1211.2 ± 21.5^b^	198.8 ± 9.5^c^
AT194	Glucose	134.9 ± 15.2^a^	96.1 ± 18.6^a^	20.8 ± 6.1^a^	140.4 ± 10.9^a^	31.9 ± 3.2^a^	152.7 ± 15.3^a^	42.1 ± 4.8^a^
	Mannitol	1834.6 ± 21.3^d^	269.1 ± 14.9^b^	60.3 ± 3.8^b^	336.9 ± 13.6^b^	51.1 ± 3.9^a^	229.1 ± 17.3^a^	58.8 ± 2.7^a^
	Inulin	391.2 ± 13.7^e^	384.4 ± 16.2^c^	52.1 ± 4.3^b^	365.1 ± 14.2^b^	31.9 ± 3.1^a^	259.6 ± 18.7^a^	42.1 ± 3.3^a^
AT195	Glucose	131.2 ± 10.3^a^	94.9 ± 10.9^a^	21.2 ± 1.7^a^	139.3 ± 11.1^a^	33.9 ± 2.8^a^	151.8 ± 17.9^a^	41.7 ± 3.5^a^
	Mannitol	892.1 ± 29.4^c^	379.6 ± 23.2^c^	116.6 ± 9.8^e^	195.1 ± 13.4^a^	44.1 ± 4.9^a^	136.6 ± 11.3^a^	45.8 ± 5.7^a^
	Sorbitol	537.9 ± 17.8^b^	189.8 ± 14.3^b^	260.7 ± 15.2^f^	334.3 ± 16.4^b^	54.3 ± 5.1^a^	151.8 ± 13.6^a^	162.6 ± 12.3^b, c^
	Lactulose	485.4 ± 10.7^b^	531.4 ± 16.2^d^	159.2 ± 11.1^g^	181.1 ± 10.6^a^	40.7 ± 4.2^a^	166.9 ± 14.2^a^	150.2 ± 11.6^b, c^

* *Hypothetical protein with molecular weights (MW) compatible with proteins involved in the metabolism of Lb. rhamnosus (Koponen et al., 2012) or deposited in Lb. rhamnosus GG genomic database (GenBank accession no. FM179322)*.

** *Different letters within columns indicate significant differences (P < 0.05)*.

A separate comment needs to be made for other four protein bands, arbitrarily labeled as P4, P5, P6, and P7, having MW between 45.7 and 21.8 kDa (Table 1). In this case, the over-expression was not detected concurrently for all the four protein bands, but it resulted affected by the specific strain cultivated in presence of specific prebiotics. P4 (corresponding to the band of 45.7 kDa) matched with the ATP-binding subunit Clpx. P5, P6, and P7 (ranging between 29.6 and 21.8 kDa) complied with dihydroorotate dehydrogenase (experimental MW of 29.6 kDa; theoretical MW 29.6 kDa), triosephosphate isomerase (experimental MW of 26.9 kDa; theoretical MW 26.9 kDa), and GrpE (experimental MW of 21.8 kDa; theoretical MW 21.8 kDa), respectively. In detail, mannitol led to the over-expression (≥ 2 fold than the CC) of P4 and P7 in GG and of P4 in AT194. Sorbitol caused the over-expression of P5, P6 and P7 in GG and of P4 and P7 in AT195. P4 resulted over-expressed in AT194 in presence of inulin, whilst lactulose caused the over-expression of P7 in AT195.

### Effect of pre-cultivation in prebiotics on the *Lb. rhamnosus* survival in simulated gastrointestinal transit

**Figure 2:**
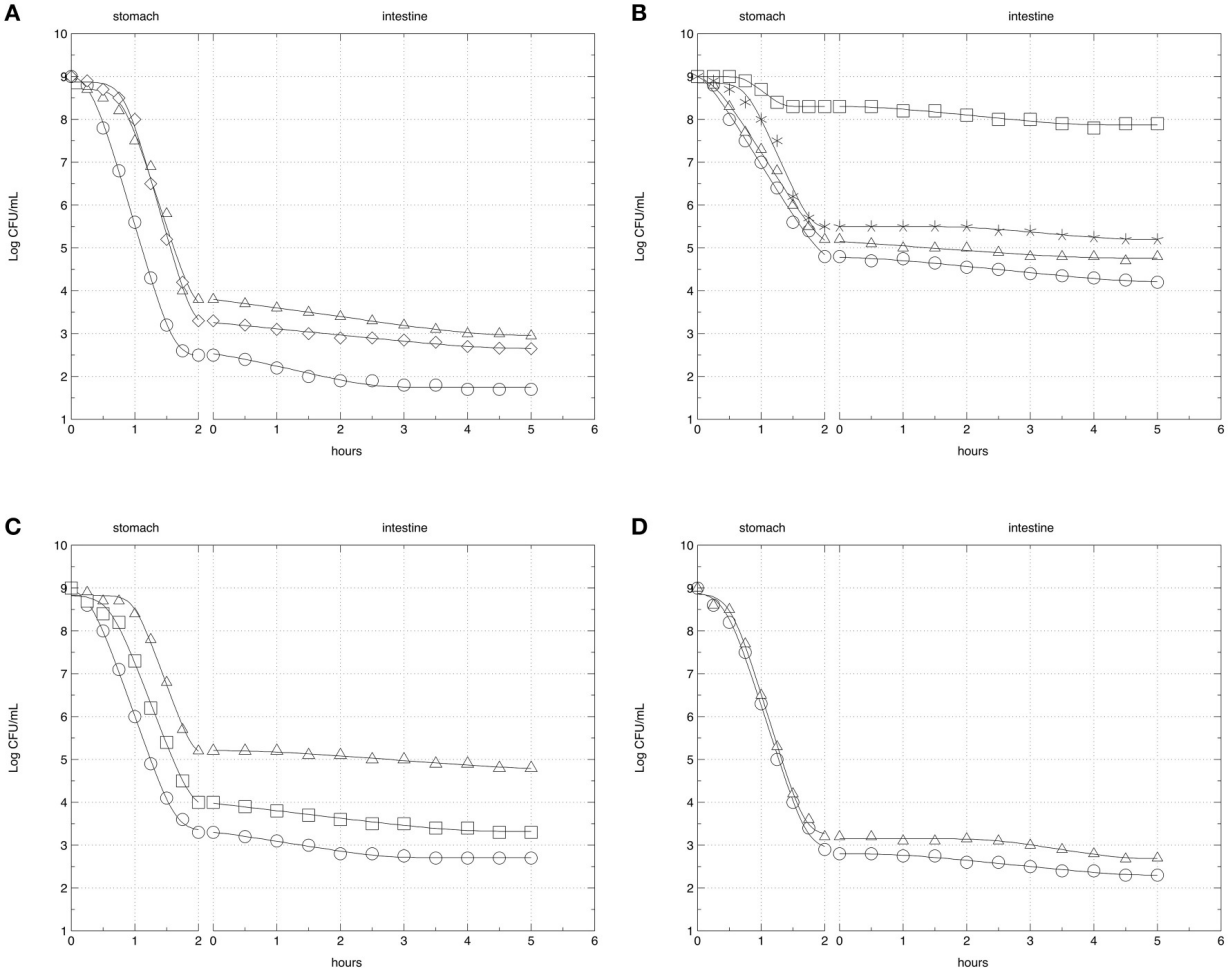
Survival curves of *Lb. rhamnosus* strains AT194 **(A)**, AT195 **(B)**, LGG **(C)**, and DSM20021 **(D)** exposed to simulated GI transit. Results refer to strains pre-cultivated in MRS broth containing glucose (circle), mannitol (triangle), inulin (diamond), sorbitol (square), or lactulose (asterisk), depending on the strain-specific ability to grow in presence of certain prebiotics.

Figure 2 shows the survival curves of *Lb. rhamnosus* strains subjected to GI stress conditions after pre-cultivation in media containing glucose or prebiotics selected on the basis of the ability to support the cell growth. The corresponding growth data are reported in Tables S1–S4. As expected, a significant decrease (*P* < 0.05) of *Lb. rhamnosus* strains was generally observed during the incubation time, and significant differences (*P* < 0.01) in the survival rates were detected between the stomach and the intestinal simulated transit. Considering the pre-cultivation in glucose (CC), a very high reduction in viable cells was highlighted when the strains were exposed to gastric stress, finding a mean decrease of about 5.7 log CFU/mL. The intestinal transit influenced the survival to a lesser extent, as shown by a mean decrease of about 0.6 log CFU/mL. Moreover, in CC significant differences were appreciated in the maximum death rate values, ranging from −5.0689 to −2.404 h^−1^ into the stomach and from −0.149 to −0.335 h^−1^ into the intestinal simulated transit.

**Figure 3:**
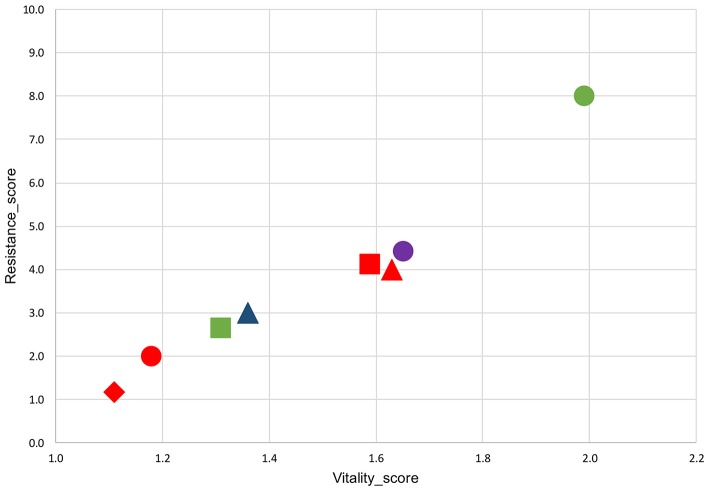
Relation between the Vitality_score and the Resistance_score of *Lb. rhamnosus* strains exposed to simulated GI transit. Results refer to strains AT194 (triangle), AT195 (circle), LGG (square) and DSM20021 (diamond) pre-cultivated in presence of mannitol (red), inulin (blue), sorbitol (green), or lactulose (purple), depending on the strain-specific ability to grow in presence of certain prebiotics.

The replacement of glucose with usable prebiotics induced in all strains the shoulder extension and the increase of the final microbial load, even if the kinetic parameters differed depending on the association prebiotic-strain. In particular, the pre-cultivation with sorbitol or mannitol enhanced the survival of AT195 or LGG, respectively. However, only through the analysis of data reported in Tables S1–S4 it was possible to ascertain a clear link existing between the shoulder extension and the increase of the microbial load (Figure 3), and this correlation was particularly remarkable at the end of the gastric simulated transit (*R*^2^ = 0.97). The highest resistance score-value was detected for the association AT195-sorbitol, to which corresponded the highest gastric-vitality-score. Contrarily, the lowest values in both resistance-score and gastric-vitality-score were detected for the association mannitol-DSM20021. Those strains having intermediate shoulder extensions in presence of specific prebiotics, had also intermediate gastric-vitality-score values, and were placed in middle positions.

### Protein up-regulation and enhancement in kinetic parameters

**Figure 4:**
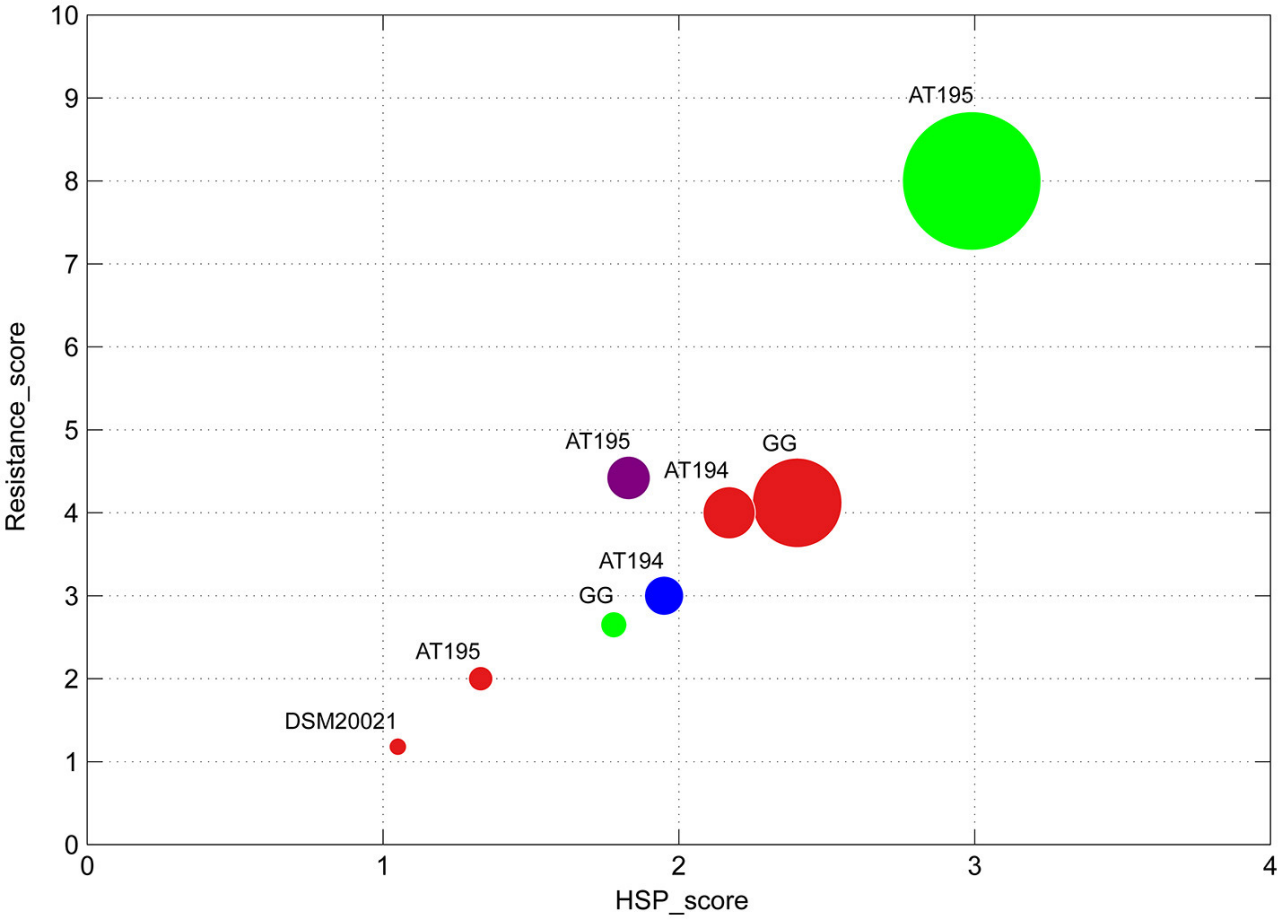
Bubble chart representing the relation between the Resistance_score, the HSP_score and the Vitality_score (bubble diameter) of *Lb. rhamnosus* strains pre-cultivated in MRS broth containing mannitol (red), sorbitol (green), inulin (blue) or lactulose (purple), and exposed to simulated GI transit.

During the simulated GI transit, a high correlation (Figure 4) was also found between the volume increase of specific protein bands (HSP_score) from specific strains cultivated in presence of certain prebiotics, the shoulder extension (Resistance_score), and the enhancement of vitality (circle diameter). Data reported in Figure 4 refer to the parameters registered at the end of the gastric phase, but results registered at the end of the entire GI transit overlap with the previous ones (data not shown). Specifically, the increase in the Resistance_score was linked to the increase in the over-expression of two stress proteins, that is, P4 (ATP-binding subunit Clpx) and P7 (GrpE). No correlation was instead found with the other protein bands discussed previously (P1-P3, P5 and P6). The strain AT195 pre-cultivated in presence of sorbitol showed the highest values in Resistance_score, HSP-score and Vitality-score. The same strain and the type strain DSM20021 exhibited the lowest values in presence of mannitol. Those strains having intermediate scores in presence of specific prebiotics were placed in middle positions.

## Discussion

In this study, we used an innovative experimental design to evaluate the influence of pre-cultivation in medium added of inulin, lactulose, mannitol, or sorbitol on the stress response of *Lb. rhamnosus* strains, including LGG, when subjected to simulated GI transit. Noteworthy, the survival of probiotic bacteria under simulated GI conditions has been extensively studied over the last decades, as reviewed by Papadimitriou et al. (2015). Differences were marked throughout the literature, depending on the strain tested and the method adopted. Some of these tests have a long history of use and are still routinely employed for their simplicity and repeatability. For this reason, we adopted a consolidated approach (Succi et al., 2014, 2017) for assessing the viability of certain *Lb. rhamnosus* strains during the simulated GI transit. Additionally, it is important to underline that the probiotic strains are actually delivered to the host already stressed due to extended fermentation periods, processing conditions and storage. This pre-stressed state may be responsible for the enhancement or the diminishment of their resistance during the passage through the host, depending on the species or strain-specific properties as well as on the conditions preceding the GI transit.

In our study, we observed that certain phenomena occurring during the growth in presence of some tested prebiotics, such as the starvation and the biochemical adaptation, were responsible for the up-regulation of specific gene products and for the resistance to GI stressors in trials carried out without prebiotics. In this field, a number of researches were made on different combinations prebiotics/probiotics, but to our knowledge, all the studies were planned to evaluate the growth, survival and performances of probiotics in presence of prebiotics. For instance, Liong and Shah (2005) tested the effect of different prebiotics, including mannitol, sorbitol and inulin, on the cholesterol removal ability of *Lactobacillus acidophilus* ATCC 4962, showing the best results in presence of mannitol and inulin. Yeo and Liong (2010) evaluated different probiotic lactobacilli and bifidobacteria for their viability in soymilk supplemented with mannitol and sorbitol, and they found out a significant improvement of soymilk features in presence of prebiotics. Several other studies were produced to describe the effect of previous carbon sources on different probiotic strains belonging to *Lactobacillus* and *Bifidobacterium* spp. (Miremadi and Shah, 2012; Sawangwan, 2015).

On the other hand, only a few studies are available on the combination of *Lb. rhamnosus* strains with lactulose, mannitol or sorbitol, whilst the literature is reach in researches on synbiotic mixtures of probiotic *Lb. rhamnosus* strains and inulin. Noteworthy, Douillard et al. (2013a) confirmed mannitol and sorbitol as fermentable substrates for *Lb. rhamnosus* strains, including LGG, whilst inulin resulted as a non-metabolizable substrate for the majority of tested *Lb. rhamnosus* strains, including LGG. Even lactulose was detected by Kontula et al. (1999) as a prebiotic fermented hardly by *Lb. rhamnosus* strains.

Our results showed that the growth of *Lb. rhamnosus* in presence of prebiotics and the subsequent events of protein up-regulation and resistance in the simulated GI transit, performed without the addition of prebiotics, were highly correlated and strain-dependent. Specifically, in all cases the assayed strains cultivated with prebiotic compounds reached final counts lower than those detected with glucose. This finding was already observed by Su et al. (2007). Moreover, the analysis of the growth kinetic parameters showed for some associations lactobacilli/prebiotics the occurrence of a growth retard that was in agreement with starvation-like phenomena already described by some Authors (Saulnier et al., 2007; Altieri et al., 2011) when lactobacilli were cultivated with prebiotics as a unique carbon source.

A further information was obtained when strains pre-cultivated with usable prebiotics, that is, those which acted as a carbon source, were subjected to simulated GI transit without prebiotics. In this case, tested strains showed a higher viability than that detected for the same strain pre-cultivated in presence of glucose. The viability was particularly evident for some associations strain/prebiotic. Interestingly we noted that prebiotics able to promote the growth of specific strains could be different from those that enhanced the viability during the GI transit. For instance, the strain AT195 showed the highest growth in presence of lactulose, even if lower than that detected with glucose, whilst the highest viability at the end of the GI transit was registered in the case of pre-cultivation with sorbitol. These findings suggest different mechanisms of response in the assayed *Lb. rhamnosus* strains, that is, the activation of response mechanisms (such as the activation of existing transport and metabolism pathways) in the case of growth with simply usable prebiotics, or the activation of resistance mechanisms in the case of pre-cultivation with specific, more hardly, usable prebiotics prior to GI stressors. In this regard, numerous studies highlighted that some prebiotics could exert positive effects on the death kinetic of lactobacilli, prolonging their viability over the time (Oliveira et al., 2009; Nazzaro et al., 2012; Adebola et al., 2014; Bevilacqua et al., 2016). However, it remains unclear how prebiotics could influence the growth and resistance of lactobacilli when exposed to harsh environments. In this context, Chen et al. (2015) tested the effect of sugar alcohols and proteins on the survival of *Lactobacillus bulgaricus* LB6 during freeze drying. They found a positive effect of sorbitol on the survival rate. Other studies, summarized in the review by Dianawati et al. (2016), evaluated the protective effect of different carbohydrates on probiotic bacteria during dehydration, during exposure to GI transit and during storage. However, all the previous mentioned studies were performed in presence of prebiotics, and no information was given on the mechanisms of response to stress factors possibly activated by prebiotics. To our knowledge, only Saulnier et al. (2007) highlighted that the presence of prebiotics influenced the expression of at least 190 genes in lactobacilli.

Our results on the survival enhancement during the GI transit, ascertained for some combinations lactobacilli/prebiotics, allowed the attribution of a key role in the response to the starvation. In fact, as also reported by other Authors (van de Guchte et al., 2002), the low substrate utilization induces kinds of starvation in lactic acid bacteria and, indirectly, the up-regulation of proteins involved into stress response to different type of conditions. In our study the over-expression of several protein bands was detected when the strains were pre-cultivated in presence of specific prebiotics. Using the approach adopted by Zotta et al. (2008), the molecular weight of the studied bands matched with proteins involved in biochemical pathways of *Lb. rhamnosus* species. Several up-regulated proteins resulted involved in different metabolic ways, such as in the carbohydrate metabolism (triosephosphate isomerase, P6) in the protein synthesis (P1, Alanyl-tRNA syntetase) in the protein secretion (P3, Protein traslocase subunit secA) and in the pyrimidine biosynthesis (P5, dihydroorotate dehydrogenase). However, the proteins reported above were found not to be related to the resistance and death kinetic parameters. Instead, the results from statistical analyses evidenced that two specific stress proteins (P4, ATP-binding subunit Clpx and P7, GrpE) were highly correlated to the strain resistance to simulated GI transit (shoulder extension) and to the enhancement in final viability. The up-regulation of stress proteins, such as proteases or chaperones is a widespread mechanism of cell protection. ClpP ATPase may function as chaperones or can associate with ClpP peptidase, forming a Clp proteolytic complex (Frees et al., 2007). GrpE is a general stress protein and its up-regulation was also associated to the starvation. Moreover, our results showed that the adaptation to adverse environments is usually associated with the synthesis of stress-response proteins, and the development of cross-resistance to various stresses (Girgis et al., 2003; Tremonte et al., 2016; Succi et al., 2017).

In conclusion, data acquired by the scientific literature highlighted that the simultaneous presence of prebiotics and probiotics ensures a higher resistance of bacterial strains to different stresses. Our results added a new information regarding the protective role of pre-cultivation with specific prebiotics on *Lb. rhamnosus* strains during the GI transit, attributing a definite role to the stress-response via cross-resistance. This fact could open a new strand in the search of strategies to enhance the survival of probiotics.

## Author contributions

MS, design of the work; analysis and interpretation of the data; drafting and revising the work; PT, interpretation of data, drafting the work and revising it critically; GP, analysis and interpretation of data; drawing up figures; statistical analysis; LT, AC, and RR: analysis of the microbial growth and interpretation of data; ES, conception of the work; drafting the work and revising it critically; RC, involved in experimental designing; coverage of all questions related to the accuracy or integrity of any part of the work are appropriately investigated and resolved.

### Conflict of interest statement

The authors declare that the research was conducted in the absence of any commercial or financial relationships that could be construed as a potential conflict of interest.
