# Supplementary material for: Pre-cultivation with Selected Prebiotics Enhances the Survival and the Stress Response of Lactobacillus rhamnosus Strains in Simulated Gastrointestinal Transit
Source: Front Microbiol. 2017 Jun 14;8:1067. doi: 10.3389/fmicb.2017.01067 (PMC5469880; doi:10.3389/fmicb.2017.01067)
Supplement: Supplementary file 1 [file Table1.PDF]

Supplementary Material

Pre-cultivation with selected prebiotics enhances the survival and the stress response of *Lactobacillus rhamnosus* strains in simulated gastrointestinal transit

Mariantonietta Succi[1], Patrizio Tremonte[1], Gianfranco Pannella[1], Luca Tipaldi[1], Autilia Cozzolino[1], Rossana Romaniello[2], Elena Sorrentino[1*], Raffaele Coppola[1]

*** Correspondence:** Elena Sorrentino: sorrentino@unimol.it

Supplementary Table

Table S1. Survival kinetic parameters registered during the simultated GI transit of AT194 pre-cultivated with fermentable prebiotics glucose, mannitol and inulin.

	**Glucose**		**Mannitol**		**Inulin**	
	Stomach	Intestine	Stomach	Intestine	Stomach	Intestine
**y_0** (Log CFU/mL)	9.0 ± 0.1	2.4 ± 0.1	8.8 ± 0.1	4.6 ± 0.0	8.9 ± 0.1	3.3 ± 0.0
**Shoulder**(h)	0.3 ± 0.0	0.1 ± 0.0	0.8 ± 0.1	1.4 ± 0.3	0.9 ± 0.1	1.5 ± 0.3
**y_end** (Log CFU/mL)	2.4 ± 0.1	1.7 ± 0.0	4.6 ± 0.2	2.9 ± 0.0	3.3 ± 0.1	2.4 ± 0.1
**µmax**(h^{-1})	-5.07 ± 0.12	-0.34 ± 0.07	-3.78 ± 0.40	-0.39 ± 0.09	-5.04 ± 0.30	-0.29 ± 0.04
**R-square:**	0.999	0.936	0.991	0.961	0.996	0.971
**SE of Fit:**	0.076	0.075	0.172	0.067	0.155	0.059

±, standard error.
