# Supplementary material for: Pre-cultivation with Selected Prebiotics Enhances the Survival and the Stress Response of Lactobacillus rhamnosus Strains in Simulated Gastrointestinal Transit
Source: Front Microbiol. 2017 Jun 14;8:1067. doi: 10.3389/fmicb.2017.01067 (PMC5469880; doi:10.3389/fmicb.2017.01067)
Supplement: Supplementary file 2 [file Table2.PDF]

Supplementary Material

Pre-cultivation with selected prebiotics enhances the survival and the stress response of *Lactobacillus rhamnosus* strains in simulated gastrointestinal transit

Mariantonietta Succi[1], Patrizio Tremonte[1], Gianfranco Pannella[1], Luca Tipaldi[1], Autilia Cozzolino[1], Rossana Romaniello[2], Elena Sorrentino[1*], Raffaele Coppola[1]

*** Correspondence:** Elena Sorrentino: sorrentino@unimol.it

Supplementary Table

Table S2. Survival kinetic parameters registered during the simultated GI transit of AT195 pre-cultivated with fermentable prebiotics glucose, mannitol, sorbitol and lactulose.

	**Glucose**		**Mannitol**		**Sorbitol**		**Lactulose**	
	Stomach	Intestine	Stomach	Intestine	Stomach	Intestine	Stomach	Intestine
**y_0** (Log CFU/mL)	9.1 ± 0.1	4.5 ± 0.0	9.0 ± 0.0	4.8 ± 0.0	8.9 ± 0.1	8.2 ± 0.0	8.8 ± 0.1	5.5 ± 0.0
**Shoulder**(h)	0.1 ± 0.0	0.6 ± 0.3	0.2 ± 0.0	0.1 ± 0.0	1.0 ± 0.1	0.7 ± 0.1	0.7 ± 0.1	2.0 ± 0.2
**y_end** (Log CFU/mL)	4.5 ± 0.4	4.2 ± 0.0	4.9 ± 0.3	4.8 ± 0.0	8.2 ± 0.1	7.3 ± 0.0	5.5 ± 0.2	5.2 ± 0.0
**μmax**(h^{-1})	-2.41 ± 0.2	-0.15 ± 0.02	-2.35 ± 0.13	-0.10 ± 0.02	-2.14 ± 0.42	-0.10 ± 0.01	-3.53 ± 0.45	-0.13 ± 0.02
**R-square:**	0.991	0.978	0.995	0.904	0.964	0.858	0.983	0.978
**SE of Fit:**	0.143	0.031	0.102	0.048	0.111	0.063	0.182	0.019

±, standard error.
