# Supplementary material for: Pre-cultivation with Selected Prebiotics Enhances the Survival and the Stress Response of Lactobacillus rhamnosus Strains in Simulated Gastrointestinal Transit
Source: Front Microbiol. 2017 Jun 14;8:1067. doi: 10.3389/fmicb.2017.01067 (PMC5469880; doi:10.3389/fmicb.2017.01067)
Supplement: Supplementary file 3 [file Table3.PDF]

Supplementary Material

Pre-cultivation with selected prebiotics enhances the survival and the stress response of *Lactobacillus rhamnosus* strains in simulated gastrointestinal transit

Mariantonietta Succi[1], Patrizio Tremonte[1], Gianfranco Pannella[1], Luca Tipaldi[1], Autilia Cozzolino[1], Rossana Romaniello[2], Elena Sorrentino[1*], Raffaele Coppola[1]

*** Correspondence:** Elena Sorrentino: sorrentino@unimol.it

Supplementary Table

Table S3. Survival kinetic parameters registered during the simultated GI transit of LGG pre-cultivated with fermentable prebiotics glucose, mannitol and sorbitol.

	**Glucose**		**Mannitol**		**Sorbitol**	
	Stomach	Intestine	Stomach	Intestine	Stomach	Intestine
**y_0** (Log CFU/mL)	9.0 ± 0.1	3.3 ± 0.0	8.8 ± 0.2	5.4 ± 0.0	8.9 ± 0.1	5.1 ± 0.0
**Shoulder**(h)	0.3 ± 0.0	0.1 ± 0.0	1.2 ± 0.1	1.8 ± 0.4	0.8 ± 0.0	0.4 ± 0.0
**y_end** (Log CFU/mL)	3.3 ± 0.1	2.7 ± 0.0	5.4 ± 0.2	4.8 ± 0.0	5.1 ± 0.2	3.3 ± 0.0
**μmax**(h^{-1})	-4.21 ± 0.12	-0.24 ± 0.02	-4.10 ± 0.35	-0.08 ± 0.02	-3.46 ± 0.20	-0.15 ± 0.05
**R-square:**	0.999	0.981	0.992	0.917	0.996	0.966
**SE of Fit:**	0.074	0.031	0.116	0.025	0.096	0.022

±, standard error.
