# Supplementary material for: Pre-cultivation with Selected Prebiotics Enhances the Survival and the Stress Response of Lactobacillus rhamnosus Strains in Simulated Gastrointestinal Transit
Source: Front Microbiol. 2017 Jun 14;8:1067. doi: 10.3389/fmicb.2017.01067 (PMC5469880; doi:10.3389/fmicb.2017.01067)
Supplement: Supplementary file 4 [file Table4.PDF]

Supplementary Material

Pre-cultivation with selected prebiotics enhances the survival and the stress response of *Lactobacillus rhamnosus* strains in simulated gastrointestinal transit

Mariantonietta Succi[1], Patrizio Tremonte[1], Gianfranco Pannella[1], Luca Tipaldi[1], Autilia Cozzolino[1], Rossana Romaniello[2], Elena Sorrentino[1*], Raffaele Coppola[1]

*** Correspondence:** Elena Sorrentino: sorrentino@unimol.it

Supplementary Table

Table S4. Survival kinetic parameters registered during the simultated GI transit of DSM20021 pre-cultivated with fermentable prebiotics glucose and mannitol.

	**Glucose**		**Mannitol**	
	Stomach	Intestine	Stomach	Intestine
**y_0** (Log CFU/mL)	8.9 ± 0.1	2.9 ± 0.0	8.6 ± 0.1	3.2 ± 0.0
**Shoulder**(h)	0.4 ± 0.1	1.0 ± 0.3	0.5 ± 0.0	2.3 ± 0.3
**y_end** (Log CFU/mL)	2.9 ± 0.2	2.3 ± 0.0	3.2 ± 0.1	2.9 ± 0.0
**μmax**(h^{-1})	-4.66 ± 0.24	-0.15 ± 0.02	-4.83 ± 0.24	-0.24 ± 0.14
**R-square:**	0.996	0.974	0.997	0.861
**SE of Fit:**	0.142	0.031	0.128	0.043

±, standard error.
